# Comparison of Steroidogenic and Ovulation-Inducing Effects of Orthosteric and Allosteric Agonists of Luteinizing Hormone/Chorionic Gonadotropin Receptor in Immature Female Rats

**DOI:** 10.3390/ijms242316618

**Published:** 2023-11-22

**Authors:** Kira V. Derkach, Ivan A. Lebedev, Irina Yu. Morina, Andrey A. Bakhtyukov, Alena S. Pechalnova, Viktor N. Sorokoumov, Veronica S. Kuznetsova, Irina V. Romanova, Alexander O. Shpakov

**Affiliations:** 1 Sechenov Institute of Evolutionary Physiology and Biochemistry, Russian Academy of Sciences, St. Petersburg 194223, Russia; derkatch_k@list.ru (K.V.D.); lebedevivan9@gmail.com (I.A.L.); bahtyukov@gmail.com (A.A.B.); sorokoumov@gmail.com (V.N.S.); nikkuznets14@gmail.com (V.S.K.); irinaromanova@mail.ru (I.V.R.); 2 Institute of Chemistry, St. Petersburg State University, St. Petersburg 199034, Russia

**Keywords:** ovarian steroidogenesis, ovulation, allosteric agonist, chorionic gonadotropin, luteinizing hormone receptor, immature female rat, vascular endothelial growth factor, metalloproteinase ADAMTS-1

## Abstract

Gonadotropins, including human chorionic gonadotropin (hCG), are used to induce ovulation, but they have a number of side effects, including ovarian hyperstimulation syndrome (OHSS). A possible alternative is allosteric luteinizing hormone (LH)/hCG receptor agonists, including the compound TP4/2 we developed, which remains active when administered orally. The aim was to study the effectiveness of TP4/2 (orally, 40 mg/kg) as an ovulation inducer in FSH-stimulated immature female rats, compared with hCG (s.c., 15 IU/rat). TP4/2 stimulated progesterone production and corpus luteum formation; time-dependently increased the ovarian expression of steroidogenic genes (*Star*, *Cyp11a1*, *Cyp17a1*) and genes involved in ovulation regulation (*Adamts-1*, *Cox-2*, *Egr-1*, *Mt-1*); and increased the content of metalloproteinase ADAMTS-1 in the ovaries. These effects were similar to those of hCG, although in some cases they were less pronounced. TP4/2, in contrast to hCG, maintained normal LH levels and increased the ovarian expression of the LH/hCG receptor gene, indicating preservation of ovarian sensitivity to LH, and did not cause a sustained increase in expression of vascular endothelial growth factor-A involved in OHSS. Thus, TP4/2 is an effective ovulation inducer that, unlike hCG, has a lower risk of OHSS and ovarian LH resistance due to its moderate stimulating effect on steroidogenesis.

## 1. Introduction

The development of effective approaches for correcting the hormonal status of the ovaries in the conditions of reproductive disorders, as well as for controlled induction of ovulation in assisted reproductive technology (ART), is one of the most important problems of modern endocrinology and reproductive medicine. The gonadotropin preparations with luteinizing hormone (LH)-like activity used for this purpose, such as the recombinant LH and human chorionic gonadotropin (hCG) and urinary hCG, have a number of disadvantages, which can cause serious side effects [1]. This is largely due to the use in the clinic of relatively high doses of hCG and LH, significantly exceeding their physiological concentrations, even in relation to the ovulatory LH peak. This is necessary to obtain a relevant effect but causes hyperactivation of LH/hCG receptors, a decrease in their expression and functional activity in ovarian cells, and, thereby, leads to a decreased sensitivity of the ovary to endogenous gonadotropins [2,3,4]. The long-term treatment of men with gonadotropins with LH-like activity also induced the resistance of steroidogenic testicular cells to LH [5]. It is also important that recombinant forms of gonadotropins differ significantly from endogenous LH circulating in the blood in their N-glycosylation pattern, which not only affects their pharmacodynamics, but also changes their selectivity in relation to intracellular signaling cascades and, thereby, modifies the cellular response [6,7]. As is known, the binding of gonadotropins to the LH/hCG receptor can lead to stimulation of various types of heterotrimeric G proteins responsible for hormonal regulation of the cAMP-dependent signaling pathway (G_s_ protein–adenylate cyclase–protein kinase A), as well as calcium-dependent signaling pathways (G_q/11_ protein–phospholipase Cβ–inositol 3,4,5-triphosphate/diacylglycerol–[Ca^2+^]_i_/protein kinase C isoforms). Along with this, when the LH/hCG receptor is activated, the process of recruitment of β-arrestins is triggered. This can lead to endocytosis of the ligand–receptor complex with its subsequent degradation or recycling, as well as activation of β-arrestin-dependent signaling pathways, including the mitogen-activated protein kinase cascade [7,8].

hCG and LH increase the expression of vascular endothelial growth factor (VEGF) and a number of other growth factors in ovarian cells, which is a consequence of hyperactivation of the LH/hCG receptor and changes in intracellular signaling bias in these cells [9]. On the one hand, activation of VEGF-dependent cascades is necessary to stimulate ovarian angiogenesis and normal folliculogenesis, but, on the other hand, an excessive increase in the expression and activity of VEGF signaling cascades leads to ovarian hyperstimulation syndrome (OHSS), a severe complication of controlled ovulation induction [10]. Replacing gonadotropins with gonadotropin-releasing hormone agonists reduces the risk of OHSS but also reduces pregnancy rates [11,12]. Somewhat better results were obtained with the combined use of GnRH and low doses of gonadotropin, but even in this case, significant side effects were shown [13]. 

All of the above makes it necessary to search for alternative ways to induce ovulation, and great expectations are associated with the low-molecular-weight heterocyclic compounds with the properties of allosteric agonists of the LH/hCG receptor [14,15,16,17]. These compounds specifically interact with the allosteric site located within the transmembrane domain of the LH/hCG receptor, while LH and hCG bind with high affinity to the orthosteric site located in the large extracellular domain of the LH/hCG receptor. The lack of overlap between the allosteric and orthosteric sites eliminates competition between gonadotropins and low-molecular-weight agonists, which prevents disruption of the response of target cells to endogenous LH in the presence of transmembrane allosteric site agonists. The most active among full allosteric agonists of the LH/hCG receptor are thieno[2,3-d]-pyrimidine derivatives, the first representatives of which were developed back in the 2000s, including the compound Org43553 [14,15,18]. Under in vivo conditions, Org43553 increased the production of steroid hormones and induced ovulation in immature female rats [15,19,20], and also induced ovulation in female volunteers [21]. 

Based on the structure of Org43553, we have developed new thieno[2,3-d]-pyrimidine derivatives with LH/hCG receptor agonist activity, which these compounds demonstrated both in vitro, in Leydig cell cultures, and in vivo, when administered to male rats [22,23,24,25]. The most active compounds TP4/2 (5-amino-N-(*tert*-butyl)-4-(3-(1-methyl-1H-pyrazole-4-carboxamido)phenyl)-2-(methylthio)thieno[2,3-d]pyrimidine-6-carboxamide) and TP03, when administered long-term to male rats with diabetes- and age-induced androgen deficiency, restored testicular steroidogenesis, normalized testosterone levels, and improved spermatogenesis [24,25]. The steroidogenic activity of these compounds in the testes suggested that, like Org43553, they may be effective for the controlled induction of ovulation in female rats, which was confirmed by studying the stimulating effect of TP03 on ovarian steroidogenesis in mature and immature female rats under conditions of its intraperitoneal administration [26,27]. At the same time, of greatest clinical interest is the oral route of administration of thieno[2,3-d]-pyrimidine derivatives since they are stable and well absorbed in the gastrointestinal tract [21,25,28]. Preliminary studies have shown that compound TP4/2, when administered orally, as an activator of ovarian steroidogenesis, is significantly superior to TP03. Based on this, we selected TP4/2 to study the effectiveness of orally administered thieno[2,3-d]-pyrimidine derivatives as ovulation inducers, while assessing a wide range of its molecular targets and markers of ovarian steroidogenesis and ovulation.

The aim of the work was to investigate the ability of the compound TP4/2, when administered orally to immature female rats, to induce ovulation. To this end, at various time intervals after administration of TP4/2, we assessed the weight of the ovaries, the number of preovulatory follicles and corpora lutea (CL), the plasma levels of progesterone, estradiol, and LH, the expression of genes involved in ovarian steroidogenesis, folliculogenesis, and ovulation, as well as the gene expression of the isoforms A and B of vascular endothelial growth factor (VEGF-A, VEGF-B), which are involved in OHSS development. Along with this, we immunochemically studied the distribution of matrix metalloproteinase with thrombospondin motifs 1 (ADAMTS-1), an important marker of terminal folliculogenesis and ovulation, in the ovaries of rats. The study was carried out in comparison with hCG, an orthosteric agonist of the LH/hCG receptor, which is currently the “gold standard” for activators of ovarian steroidogenesis and is widely used in ART for ovulation induction [1]. 

As a result of our studies, it was shown for the first time that the allosteric agonist of the LH/hCG receptor, which we developed, when administered orally to follicle-stimulating hormone (FSH)-stimulated immature female rats, stimulates ovarian steroidogenesis and induces the formation of CL, and its effects are similar to those of hCG. At the same time, unlike hCG, TP4/2 does not reduce the sensitivity of the ovaries to stimulation by endogenous LH, since it weakly affects the plasma LH level and maintains high FSH-stimulated expression of the LH/hCG receptor gene in the ovaries. It also increases the ovarian expression of VEGF-A to a significantly lower degree, which prevents the risks of developing OHSS, and this is due to the more moderate stimulatory effect of TP4/2 on ovarian steroidogenesis. Both these features, as well as its effectiveness when administered orally, allow us to consider TP4/2 as a prototype for effective and safe ovulation inducers.

## 2. Results

### 2.1. Induction of Ovulation in Immature Female Rats, the Ovarian Weight and Body Weight/Ovarian Weight Ratio in Animals and the Effect of Treatment with TP4/2 and hCG

**Figure 1:**
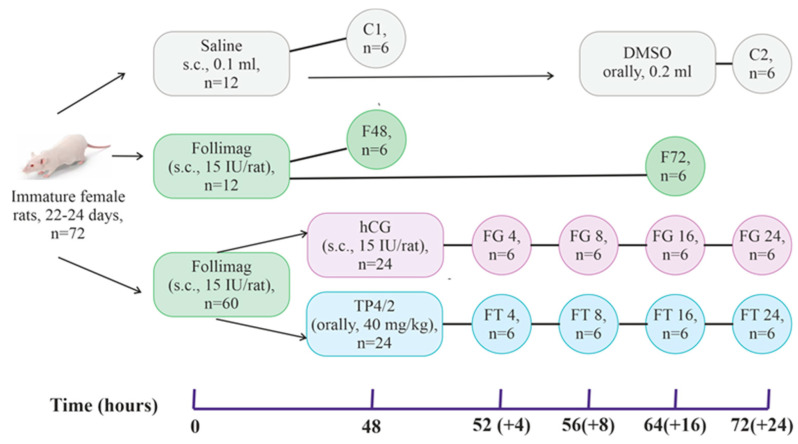
Experimental design for ovulation induction in “Follimag”-stimulated immature female Wistar rats using orthosteric (hCG) and allosteric (TP4/2) agonists of the LH/hCG receptor. The experimental design and the studied groups of immature female rats are presented, and the time points for the administration of hormonal agents and decapitation of the animals are indicated. First, immature female rats were subcutaneously injected with “Follimag” at a single dose of 15 IU/rat (except for control groups), and after 48 h, 24 rats were treated with TP4/2 (single administration, orally, 40 mg/kg of body weight) and the same number of animals were treated with hCG (single administration, s.c., 15 IU/rat). Subsequently, at certain intervals (4, 8, 16, and 24 h after the administration of each LH/hCG receptor agonist), the rats were decapitated, and the studied parameters were analyzed. Two groups of animals treated with “Follimag” did not receive LH/hCG receptor agonists and were decapitated 48 and 72 h after treatment with this FSH drug (the groups F48 and F72, respectively).

To stimulate folliculogenesis, we used “Follimag”, a gonadotropin with FSH-like activity, which was administered subcutaneously to immature female rats (aged 22–24 days) in a single dose of 15 IU/rat. At 48 h after treatment with “Follimag”, the animals were treated with ovulation inducers, such as TP4/2 (orally, 40 mg/kg) and hCG (s.c., 15 IU/rat) (Figure 1). The control rats received DMSO, a TP4/2 solvent, which was administered orally. Twelve groups of animals (each with six rats) were studied (Figure 1): (1–2) control rats (groups C1 and C2), which instead of “Follimag” were administered its solvent at the same time and were removed from the experiment 48 h (C1) and 72 h (C2) after its start; (3–4) rats that were injected with “Follimag” and then withdrawn from the experiment at 48 or 72 h (groups F48 and F72); (5–8) rats, which were injected with “Follimag”, then, 48 h later, treated with hCG (15 IU/rat, s.c.), and decapitated 4, 8, 16, and 24 h after gonadotropin administration (groups FG4, FG8, FG16, and FG24); and (9–12) rats, which were injected with “Follimag”, then, 48 h later, treated with TP4/2 (40 mg/kg, orally), and decapitated 4, 8, 16, and 24 h after TP4/2 administration (groups FT4, FT8, FT16, and FT24). 

In the animals, the total mass of the left and right ovaries and its ratio to body weight were assessed (Table 1). The treatment of animals with “Follimag” (15 IU/rat, s.c.), an FSH preparation that stimulates folliculogenesis, leads to an increase in the ovary weight and the ratio of ovary weight to body weight compared to control animals. TP4/2 (40 mg/kg, orally) and hCG (15 IU/rat, s.c.) further increased the ovary weight/body weight ratio not only in comparison with control animals (in all groups treated with TP4/2 and hCG), but also in comparison with the F48 and F72 groups (in the FT16 and FT24 groups and in all studied groups with hCG treatment) (Table 1). 

### 2.2. The Number of Tertiary and Preovulatory Follicles and Corpus Luteum in Immature Female Rats, and the Effect of Treatment with TP4/2 and hCG 

**Figure 2:**
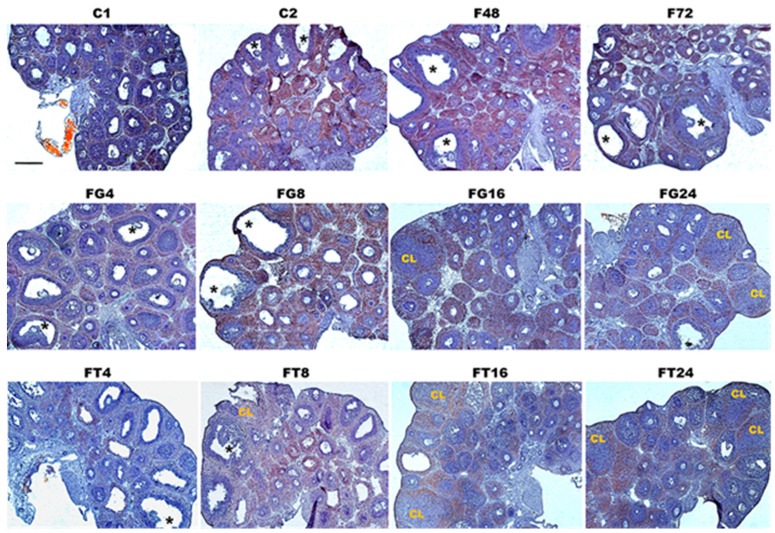
Cross-sections of the ovaries of immature female rats, 4–24 h after treatment with TP4/2 and hCG. The thickness of the sections was 10 µm. Staining was carried out with Sudan-3 and hematoxylin. Asterisks—preovulatory follicles, and CL—corpus luteum. In all photographs, the scale is 400 µm (as in the photo for group C1). Designations of animal groups: C1—control rats, corresponding to the start of animal treatment with LH/hCG receptor agonists (48 h after the beginning of the experiment); C2—control rats, corresponding to the end of the experiment; F48 and F72—rats treated with “Follimag” (15 IU/rat, s.c.) 48 or 72 h before being withdrawn from the experiment; FG4, FG8, FG16, and FG24—rats that were treated with “Follimag” and after 48 h were treated with hCG (15 IU/rat, s.c.), after which they were decapitated (after 4, 8, 16, and 24 h, respectively); FT4, FT8, FT16, and FT24—rats that were treated with “Follimag” and after 48 h were treated with TP4/2 (40 mg/kg, orally), after which they were decapitated (after 4, 8, 16 and 24 h, respectively).

To study the ability of TP4/2 and hCG to influence folliculogenesis and induce ovulation in immature female rats, a morphological analysis of the ovaries was performed, assessing the number of tertiary (antral) and preovulatory follicles, as well as the number of CL in each ovary. In the C1 and C2 groups, the primordial, primary, secondary, and tertiary follicles, with a predominance of the tertiary follicles, were identified, and single preovulatory follicles were also detected; in the C2 group, the preovulatory follicles were clearly visible on the surface of the ovaries (Figure 2). In the F48 and F72 groups, an increase in the area of the ovaries was observed, mainly due to an increase in the volume of tertiary follicles compared to the C1 and C2 groups, without a significant increase in their number, and the number of preovulatory follicles was also observed to increase over time. Meanwhile, in these groups, ovulated follicles and CL were not detected (Figure 2, Table 2). The FG4 and FT4 groups showed an increase in ovarian size due to an increase in the number of tertiary (antral) and preovulatory follicles and an increase in the volume of preovulatory follicles. The number of preovulatory follicles increased significantly in comparison with the control group and the F48 group. Single ovulated follicles were also observed, but no CL were detected (Figure 2, Table 2). The FG8 and FT8 groups showed a decrease in the number of preovulatory follicles, while the number of ovulated follicles, on the contrary, increased. In addition, single CL have been identified in the ovaries of some animals. At 16 and 24 h after treatment with LH/hCG receptor agonists, a significant decrease in the number of preovulatory follicles was observed in the ovaries of female rats, which was accompanied by the formation of a large number of CL. At the same time, if in the case of hCG, the maximum number of CL was demonstrated already 16 h after treatment with gonadotropin (in the FG16 group), while in the case of treatment with TP4/2, the maximum number of CL was achieved only after 24 h (in the FT24 group) (Figure 2, Table 2).

### 2.3. Plasma Levels of Estradiol, Progesterone, and Luteinizing Hormone in Immature Female Rats, and the Effect of Treatment with TP4/2 and hCG

In the ovulatory cycle, the trigger for follicle maturation in the later stages of folliculogenesis and the inducer of ovulation is a change in the levels and ratios of steroid hormones. This is based on an increase in estradiol levels in response to ovarian stimulation by LH and FSH and a decrease in estradiol levels several hours after the preovulatory LH peak, resulting in the estradiol peak being replaced by a progesterone peak [29,30]. In accordance with this, the dynamics of changes in the concentrations of these hormones in the blood of immature female rats treated with LH/hCG receptor agonists were assessed. The level of estradiol 4 h after treatment with TP4/2 and hCG was increased both in comparison with control (C1, C2) and “Follimag”-treated (F48, F72) rats, but subsequently decreased significantly, to a greater extent in the case of treatment of rats with gonadotropin (Table 3). In the FG16 and FT16 groups, the concentration of estradiol was 15 and 26% of that in the FG4 and FT4 groups, respectively (Table 3). Progesterone concentrations increased with TP4/2 and hCG treatment, in both cases reaching the maximum 16 h after treatment (Table 3). At the same time, the stimulating effect of hCG on progesterone levels exceeded that of TP4/2, as shown when comparing the groups FG8 and FT8 and the groups FG16 and FT16 (Table 3). In addition, in the FG8 group, the level of progesterone was significantly higher than in the control, which preceded the formation of a significant number of CL in the ovaries, demonstrated 16 h after treatment of animals with gonadotropin. 

Since the treatment of female rats with relatively high doses of LH/hCG receptor agonists can negatively affect the production of endogenous LH, the content of this gonadotropin in the blood of animals was assessed. In rats treated only with “Follimag”, after 48 h (F48), the plasma LH level was significantly reduced, but after 72 h (F72), it was restored to control values (Table 3). The administration of hCG and TP4/2 at an early stage (after 4 h) led to a significant increase in LH levels in comparison with the F48 group, to its values in the control. At the same time, later in the case of hCG, a sharp decrease in LH levels was observed, especially in the FG8 group, while in the case of TP4/2, the LH concentration in the blood was indistinguishable from that in the control and F72 groups (Table 3). 

### 2.4. The Expression of Steroidogenic Genes and the LH/hCG Receptor Gene in the Ovaries of Immature Female Rats, and the Effect of Treatment with TP4/2 and hCG

Expression of the LH/hCG receptor gene in the ovaries was significantly increased in the F48 and F72 groups treated with “Follimag” alone, as well as in the groups of rats 4 h after their treatment with LH/hCG receptor agonists (Table 4). At the same time, 8–24 h after treatment with hCG and TP4/2, the dynamics of changes in the Lhcgr gene expression differed significantly. In the case of hCG treatment, it sharply decreased, while in the case of TP4/2 treatment, on the contrary, Lhcgr gene expression remained at a level significantly higher than its control values (Table 4). 

The gene expression of the StAR protein, responsible for the transport of cholesterol into mitochondria, the rate-limiting stage of ovarian steroidogenesis, was significantly increased when rats were treated with both LHR agonists, and in the case of hCG treatment, the level of expression of the Star gene was 12–17 times higher than its values in the control and “Follimag”-treated animals. In the case of TP4/2, a significant increase in Star gene expression was observed 4 h after treatment (+1314%), but subsequently (8–24 h) the stimulatory effect of TP4/2 was less pronounced, although the Star gene expression remained higher than in the control and F48 groups (but not in comparison with the F72 group) (Table 4). Similar dynamics of the stimulating effect of TP4/2 and hCG were also shown in relation to the expression of the Cyp11a1 gene, encoding cytochrome P450_scc_ (CYP11A1), which converts cholesterol into pregnenolone. In this case, the stimulating effect of hCG was also more pronounced than that of TP4/2, as shown when comparing the groups FG16 and FT16 and the groups FG24 and FT24 (Table 4). 

The expression of the Cyp17a1 gene was increased in all studied hCG- and TP4/2-treated groups, with a maximum at 8 and 16 h after treatment (Table 4). This gene encodes cytochrome P450c17 (CYP17A1), which catalyzes the conversion of pregnenolone to 17-OH-pregnenolone and then to dehydroepiandrosterone (DHEA) or the conversion of progesterone to 17-OH-progesterone and then to androstenedione. The expression of the Cyp19a1 gene, encoding aromatase (cytochrome P450c19), which converts androgens to estrogens, was increased significantly 4 h after hCG treatment in the FG4 group, but did not change significantly in the FT4 groups. In the FT8 group, it decreased to the control level and then did not change 16 and 24 h after treatment with TP4/2. At the same time, the expression of the Cyp19a1 gene was considerably suppressed in the FG8, FG16, and FG24 groups, amounting to no more than 14% of aromatase gene expression in the control (Table 4). 

The data obtained indicate that hCG and TP4/2 enhance the expression of the steroidogenic genes Star, Cyp11a1, and Cyp17a1, while in relation to the aromatase gene (Cyp19a1), the increased level of expression of this gene persists after 4 h, but then either normalizes (TP4/2) or decreases below control values (hCG).

### 2.5. The Expression of the Genes Encoding the Isoforms A and B of Vascular Endothelial Growth Factor in the Ovaries of Immature Female Rats, and the Effect of TP4/2 and hCG Treatment

Since VEGF, which is a target for gonadotropins with LH activity in the ovaries of ovulating rats, is directly involved in the implementation of the angiogenic and proinflammatory effects that mediate ovulation, and, along with this, is responsible for the possible development of OHSS [31,32], we assessed the gene expression of two main isoforms of this factor, VEGF-A and VEGF-B, regulating VEGF-dependent cascades in the ovaries. In the F48 and F72 groups, the expression of the Vegf-a gene did not change, while the expression of the Vegf-b gene, on the contrary, was significantly suppressed (Table 5). Treatment of rats with hCG led to a significant increase in Vegf-a gene expression 4–16 h after treatment, but then the stimulating effect of gonadotropin was decreased (Table 5). In the case of TP4/2, 4 h after treatment with an allosteric agonist, the expression of the Vegf-a gene increased significantly compared to the F48 group (but not to the control), and subsequently in the FT8–FT24 groups, the level of expression of the Vegf-a gene did not differ from the control and “Follimag”-treated groups (Table 5). It is interesting to note that the nature of the LH/hCG receptor agonist had little effect on the expression of the Vegf-b gene, which, as in the F48 and F72 groups, remained at a very low level when rats were treated with hCG and TP4.2 (Table 5). 

### 2.6. The Expression of Genes Involved in Late Stages of Folliculogenesis and Ovulation in the Ovaries of Immature Female Rats and the Effect of TP4/2 and hCG Treatment 

To study the effect of LH/hCG receptor agonists on terminal folliculogenesis, ovulation, and CL formation, four marker genes were selected that are expressed in ovarian granulosa during different phases of the ovulatory cycle [33,34]. The expression of the genes encoding cyclooxygenase-2 (*Cox-2*) and early growth response protein-1 (*Egr-1*) reached maximum values 4 h after treatment with TP4/2 and hCG, and was many times higher than the expression of these genes in the ovaries of control and “Follimag”-treated animals (Table 6). At the same time, after 8 h, a sharp decrease was observed in the expression of the *Cox-2* and *Egr-1* genes, which 24 h after treatment had values close to control values (Table 6). 

The expression of the *Mt-1* gene, encoding metallothionein-1, on the contrary, increased in the time interval from 4 to 24 h, reaching a maximum in the FT24 and FG24 groups, and the stimulating effect of gonadotropin was more pronounced in comparison with that of TP4/2 (Table 6). The expression of the *Adamts-1* gene encoding the matrix metalloproteinase, ADAMTS-1, was increased at all time points but reached maximum values at 8 h after treatment with TP4/2 and hCG (Table 6). Thus, both LH/hCG receptor agonists increased the expression of the *Cox-2*, *Egr-1*, *Mt-1,* and *Adamts-1* genes involved in the regulation of terminal folliculogenesis and ovulation, and the observed temporal and quantitative changes in the expression of these genes were generally consistent with those during the gonadotropin-induced ovulatory cycle in rats.

### 2.7. Immunohistochemical Analysis of the Distribution of Metalloproteinase ADAMTS-1 in the Ovaries of Immature Female Rats and the Effect of Treatment with TP4/2 and hCG

The main function of the metalloproteinase ADAMTS-1 is the degradation of the extracellular matrix, which is critical for normal terminal folliculogenesis and determines the ability of the oocyte to be fertilized by sperm [35,36,37,38]. Based on this, the distribution of ADAMTS-1 in the ovaries of female rats at various time points after treatment with TP4/2 and hCG was studied using an immunohistochemical approach with polyclonal anti-ADAMTS-1 antibody. 

**Figure 3:**
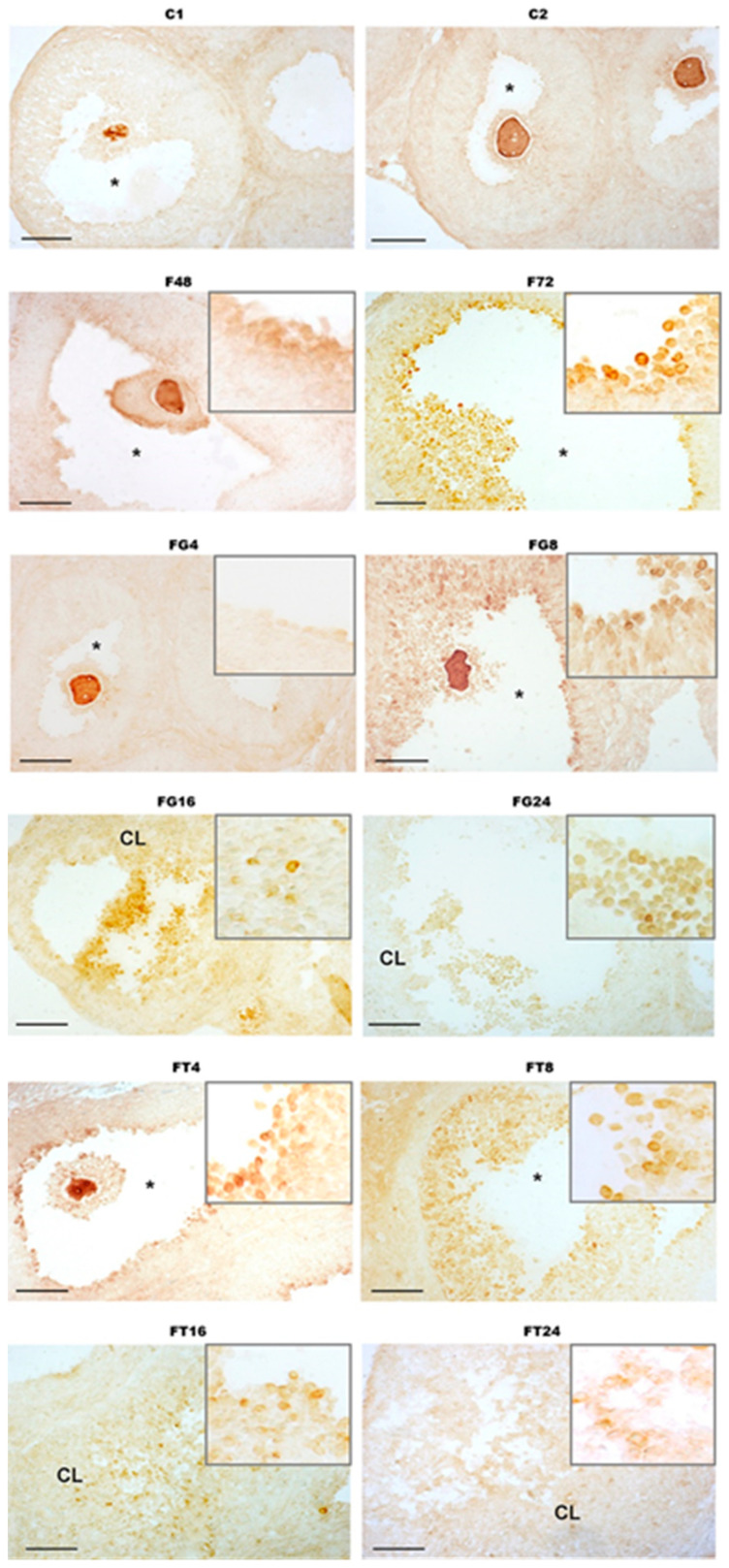
Immunohistochemical detection of metalloproteinase ADAMTS-1 on cross sections of the ovaries of immature female rats treated with “Follimag” and LH/hCG receptor agonists. The thickness of the sections was 10 µm. In all full-size photographs, the scale is 100 µm. Asterisks—preovulatory follicles, and CL—corpus luteum. The framed photographs show representative sections of the ovaries from the full-size photographs at higher magnification. Designations of animal groups: C1—control rats, corresponding to the start of animal treatment with LH/hCG receptor agonists (48 h after the beginning of the experiment); C2—control rats, corresponding to the end of the experiment; F48 and F72—rats treated with “Follimag” (15 IU/rat, s.c.) 48 or 72 h before being withdrawn from the experiment; FG4, FG8, FG16, and FG24—rats that were treated with “Follimag” and after 48 h were treated with hCG (15 IU/rat, s.c.), after which they were decapitated (after 4, 8, 16, and 24 h, respectively); FT4, FT8, FT16, and FT24—rats that were treated with “Follimag” and after 48 h were treated with TP4/2 (40 mg/kg, orally), after which they were decapitated (after 4, 8, 16, and 24 h, respectively).

In control female rats, no ADAMTS-1-immunopositive cells were detected in the wall of maturing follicles (Figure 3). In the F48 group, a weak immunoreactivity to ADAMTS-1 (+/−) was detected in the follicular cells of the preovulatory follicles, while in the F72 group, a significant number of ADAMTS-1-immunopositive cells were identified in both the follicle wall and around the maturing oocyte in the corona radiata cells (+) (Figure 3). In female rats treated with hCG, the maximum immunoreactivity was observed in the FG8 group, where ADAMTS-1 was detected in the cells of preovulatory and ovulated follicles (++). In the FG16 group, ADAMTS-1 immunopositivity was shown in the follicular cells of ovulated follicles and in residual follicular cells in the CL (+). A similar level of immunoreactivity was shown for the FG4 group, where ADAMTS-1 expression was detected in some follicular cells in preovulatory follicles (+). A relatively low level of ADAMTS-1 expression was demonstrated in the FG24 group in the follicular cells of ovulated follicles and in residual follicular cells in the CL (+/−) (Figure 3). As in the case of hCG treatment, in sections of the ovaries of TP4/2-treated female rats, the maximum immunoreactivity to ADAMTS-1 was detected in the FT8 group, 8 h after treatment with TP4/2 (++). The FT4 and FT16 groups showed significant but less pronounced immunoreactivity to ADAMTS-1 (+). In the FT4 group, ADAMTS-1 was detected in some follicular cells in preovulatory follicles, mainly in the cells surrounding the oocyte, while in the FT16 group, this protein was immunochemically detected mainly in the wall of ovulated follicles and in the CL. In the FT24 group, single ADAMTS-1-immunopositive cells were detected in the wall of ovulated follicles and in the CL (+/−) (Figure 3). Thus, the amount of ADAMTS-1 protein, as well as the expression of the *Adamts-1* gene, was maximum at those stages (the FT8 and FG8 groups) that precede the rupture of preovulatory follicles and the formation of the CL. 

## 3. Discussion

The main trigger of ovulation in humans and animals is a sharp, multiple increase in the level of LH in the blood, which leads to activation of the LH/hCG receptor–G_s_ protein–adenylate cyclase–cAMP–protein kinase A signaling pathway in mural granulosa cells and triggers a cascade of biochemical reactions that mediate oocyte maturation, release of the oocyte from the follicle, and formation of the CL. Gonadotropin preparations, including hCG, are widely used as ovulation inducers in ART, but they have a number of side effects and limitations, which motivates the search and development of alternative ovulation inducers, including those based on low-molecular allosteric agonists of the LH/hCG receptor. Among the possible candidates is the compound TP4/2, a thieno[2,3-d]-pyrimidine derivative, which we previously developed to stimulate testicular steroidogenesis [23,24]. When administered to male rats, this compound was active not only when administered intraperitoneally and subcutaneously, but also when administered orally, being stable in the gastrointestinal tract. 

We have shown that when the compound TP4/2 was orally administered at a dose of 40 mg/kg to immature female rats, previously stimulated with FSH, an increase in the relative weight of the ovaries and induction of ovulation occurred. Already 4 h after treatment with TP4/2, an increase in the number of preovulatory follicles was observed, and after 8–24 h an increase in the number of ovulated follicles and CL was detected with a sharp decrease in the number of preovulatory follicles. Similar changes in folliculogenesis were shown for hCG, the “gold” standard of ovulation inducers used in ART, with the only difference being that the number of CL with hCG treatment reached a maximum after 16 h, while in the case of TP4/2 this occurred slightly later, after 24 h (Figure 2, Table 2). Thus, TP4/2 and hCG are both effective in inducing ovulation and stimulating CL formation, although there are some differences between the drugs in the pharmacodynamics of their stimulating effects on folliculogenesis in immature female rats. Previously, other authors have shown that the compound Org43553, also belonging to the class of thieno[2,3-d]-pyrimidine derivatives, is capable of inducing ovulation in immature and cyclic female rats when administered orally at a dose of 50 mg/kg [19,20], as well as in female volunteers of reproductive age with oral administration of the drug at a minimum dose of 300 mg [21]. We have previously shown the stimulating effect of another thieno[2,3-d]-pyrimidine derivative, TP03, on the induction of ovulation in immature and cyclic rats, but the drug was administered intraperitoneally since its effectiveness was significantly reduced when administered orally [26,27]. 

In our study, we not only demonstrated the ovulation-inducing effect of TP4/2, but also for the first time, with respect to low-molecular-weight LH/hCG receptor agonists, we studied in detail the various stages of terminal folliculogenesis after administration of TP4/2 to animals, assessing both levels steroid hormones and follicle morphology, as well as the expression of the main markers of ovarian steroidogenesis and the ovulatory cycle, including cyclooxygenase-2 and metalloproteinase ADAMTS-1, which are functionally important for the normal course of ovulation.

As numerous clinical and experimental studies have shown, an increase in FSH levels at the end of the follicular phase leads to an increase in estrogen production. Estrogen synthesis occurs in ovarian granulosa cells and is catalyzed by the enzyme aromatase (cytochrome CYP19A1), which converts androgens that come from theca cells, in response to their stimulation by LH, into estrogens, and both of these processes are LH- and FSH-dependent [39,40]. Subsequently, increased levels of estradiol, through a negative feedback mechanism, reduce the production of FSH by the anterior pituitary gland and induce a peak in endogenous LH, resulting in a switch of ovarian steroidogenesis to the synthesis of progesterone in granulosa cells, which leads to a peak in progesterone levels and the formation of the CL secreting this hormone [29,40]. One of the mechanisms for switching steroidogenesis from the synthesis of estradiol to the synthesis of progesterone is a decrease in the expression of the aromatase gene and an increase in the expression of genes for steroidogenic proteins, including the StAR protein, responsible for the transport of cholesterol into mitochondria, and the cytochrome CYP11A1, which catalyzes the conversion of cholesterol to pregnenolone [39]. It has been established that the LH peak, through activation of the LH/hCG receptor–G_s_ protein–adenylate cyclase–cAMP–protein kinase A signaling pathway, causes increased degradation of aromatase mRNA and simultaneously reduces the level of transcription of the gene for this enzyme [41].

In agreement with the above, we have shown that the level of estradiol in the blood of rats in the FG4 and FT4 groups significantly increased both in comparison with the control and with rats treated with “Follimag”. At the same time, subsequently (8–24 h after treatment with TP4/2 or hCG), the concentration of estradiol decreased significantly, which was associated with a significant decrease in aromatase expression in the ovaries. In the groups treated with TP4/2 and hCG, an increase in the level of progesterone in the blood was observed (with a maximum after 16 h), as well as a preceding increase in the expression of steroidogenic genes encoding the transport protein StAR, cytochrome CYP11A1, as well as cytochrome CYP17A1, which catalyzes the conversion of pregnenolone and progesterone into their 17-OH derivatives. Increased expression of these enzymes was positively correlated with the ability of both LH/hCG receptor agonists to increase progesterone production. Despite the qualitative similarity of the effects of TP4/2 and hCG on ovarian steroidogenesis, they were quantitatively different. The stimulating effect of TP4/2 on progesterone production and on the expression of the *Star* and *Cyp11a1* genes was less pronounced than in the case of hCG. Despite the more moderate stimulation of aromatase gene expression in the FT4 group as compared to the FG4 group, the expression of this gene in the ovaries of rats 8–24 h after TP4/2 treatment did not differ from that in the control, while in the corresponding groups with hCG treatment, it was significantly lower than in both the control and TP4/2-treated groups. 

As early as 40 years ago, it was found that at the stage preceding the induction of ovulation, the expression of LH/hCG receptors in granulosa cells significantly increases, which makes them more sensitive to endogenous LH and thereby ensures the intensity of LH activation necessary for the normal functioning of ovulation-dependent cascades [42]. Subsequently, it was shown that both FSH, mainly through the activation of cAMP-dependent signaling, and a number of other factors (interleukin-6, EGR-1, and others) are involved in stimulating the expression of the *Lhcgr* gene [43,44,45,46,47]. There is evidence that in mature female mice, medium doses of a GnRH agonist highly effectively stimulate the expression of the LH/hCG receptor in the ovaries, increasing the level of progesterone in the blood and the expression of genes encoding the StAR protein and 3β-hydroxysteroid dehydrogenase [48]. 

We observed an increase in the expression level of the *Lhcgr* gene in the ovaries of rats that received only “Follimag”, as well as in groups of “Follimag”-treated animals 4 h after administration of LH/hCG receptor agonists (Table 4). However, the effects of TP4/2 and hCG on *Lhcgr* gene expression 8–24 h after their administration differed significantly. Gonadotropin reduced the expression of the *Lhcgr* gene to the control level, while in the FT8–FT24 groups a relatively high expression of this gene remained, corresponding to its level in the F48 and F72 groups, which indicates the absence of a suppressive effect of the allosteric agonist TP4/2 on the sensitivity of ovarian cells to endogenous LH (Table 4). This may be due to the more modest stimulatory effect of TP4/2 on the receptor, as illustrated by the magnitude of its stimulatory effects on progesterone production and steroidogenic gene expression compared to hCG, as well as the altered signaling bias triggered by this small molecule agonist. In this regard, it should be noted that upon long-term administration to male rats, TP4/2 and other thieno[2,3-d]-pyrimidine derivatives developed by us had a relatively weak effect on the expression of the *Lhcgr* gene in the testes, while hCG in this case significantly reduced it [24,28,49]. We and other authors have shown that allosteric agonists based on thieno[2,3-d]-pyrimidine structure are highly specific for G_s_ proteins and cAMP-dependent signaling pathways, but have little effect on the activity of G_q/11_ proteins and phospholipase Cβ [15,23]. However, the possible influence of signaling bias induced by various LH receptor agonists on the expression of this receptor gene has not been studied and requires additional research. The fact that the expression of the *Lhcgr* gene during treatment with TP4/2 remains at a relatively high level may provide a more favorable course of the luteal phase of the ovarian cycle since in the middle of this phase there is a repeated increase in LH/hCG receptor expression, initiated by the progesterone peak [50,51,52]. This occurs after a transient, highly pronounced decrease in *Lhcgr* gene expression in response to an acute surge of endogenous LH that induces ovulation [46,53], which has also been shown in female rats [54,55]. 

One of the most severe complications of ART when using gonadotropins with LH activity as ovulation inducers is the development of OHSS [56,57]. Among the reasons for the development of this syndrome is the increased secretion of VEGF by luteinized granulosa cells in response to the ovulatory peak of LH or the administration of exogenous hCG, and the greatest risk of developing OHSS is characteristic of patients with polycystic ovary syndrome [31,32]. Administration of hCG to experimental animals leads to an increase in the production of VEGF, activation of the VEGF receptor, and triggering of VEGF-dependent signaling cascades. The result of this is a significant increase in the production of vasoactive substances, including cyclooxygenase-2, and an increase in the permeability of the vascular wall, which provokes the development of OHSS [58,59,60,61]. 

We showed a hCG-induced sustained increase in the expression of the gene encoding VEGF-A, which is a key factor in ovarian angiogenesis, in the ovaries of immature female rats. This increase persisted at all intervals studied and weakened after 24 h. At the same time, the TP4/2 transiently increased the expression of the *Vegf-a* gene, only 4 h after treatment, and subsequently this indicator did not differ from control values. Along with epidermal growth factor, interleukin-8, basic fibroblast growth factor, and tumor necrosis factor-α, VEGF-A is one of the key angiogenic factors, the overproduction of which leads to OHSS [62,63]. As a result, the transient increase in *Vegf-a* expression, which disappeared 8 h after TP4/2 treatment and was associated with a less pronounced increase in ovarian weight compared with hCG, may indicate a low risk of developing OHSS with the use of this low-molecular-weight agonist. This can be considered an important advantage of TP4/2 for its possible use in ART. It should also be noted that previously other authors did not detect signs of OHSS when using another thieno[2,3-d]-pyrimidine derivative, Org43553, both in experiments with animals and when inducing ovulation in female volunteers [19,20,21]. 

It should be noted that in all groups treated with LH/hCG receptor agonists, the expression of another VEGF isoform, VEGF-B, was significantly suppressed. According to recent studies, VEGF-B is involved in the control of lipid homeostasis and insulin production [64] and acts as an activator of antioxidant cascades [65]. Besides this, VEGF-B acts as an antagonist of VEGF-A in the regulation of angiogenesis, making it an important target for the development of anticancer drugs [66,67,68]. VEGF-B, like VEGF-A, binds to vascular endothelial growth factor receptor-1 (VEGFR-1), which has very low kinase activity but is not able to activate the VEGF receptor type 2, which plays a key role in angiogenesis activation and is the main target of VEGF-A [69]. There is evidence that the VEGFR-1 receptor may also function as a decoy receptor for VEGF-A, thereby reducing angiogenesis [70,71]. Thus, decreased VEGF-B expression may also contribute to increased angiogenesis, reducing the inhibitory effect of this growth factor isoform on VEGF-A-induced angiogenesis. 

There are a large number of factors that regulate the main stages of ovulation in response to the preovulatory LH surge, which, thereby, are markers of the normal course of this process in humans and animals [33,72,73]. These factors include early growth response protein-1 (EGR-1), cyclooxygenase-2 (COX-2), matrix metalloproteinase containing multiple thrombospondin repeats-1 (ADAMTS-1), and metallothionein-1 (MT-1). 

The EGR-1 protein, expressed in various types of ovarian cells, including granulosa cells [74], is a zinc finger transcriptional factor that, depending on the cell type, regulates the expression of a large number of genes, including pro-inflammatory and angiogenic factors, as well as those controlling folliculogenesis, ovulation, and CL formation [75]. In knockout mice for the *Egr-1* gene, folliculogenesis was impaired and the animals became infertile [76,77]. Its expression increases sharply immediately after the ovulatory LH peak [33,78], and it is closely related to the expression of VEGF and fibroblast growth factor (FGF), indicating the involvement of EGR-1 in ovarian angiogenesis and, in an unfavorable scenario, in the development of OHSS [79]. We showed a 40-fold increase in the expression of the *Egr-1* gene in the ovaries of rats in the FG4 and FT4 groups, 4 h after treatment with hCG and TP4/2, compared to the control group (Table 6). This coincided with an increase in the number of preovulatory follicles and anticipated the CL formation, but, on the other hand, it was associated with an increase in the gene expression of VEGF-A, which, as noted above, is a risk factor for OHSS (Table 2, Table 5 and Table 6). After 24 h, the *Egr-1* gene expression was significantly lower in the FT24 group than in the FG24 group, which correlated well with lower expression of the gene encoding VEGF-A in the FT24 group, indicating a possible contribution of EGR-1 to the more modest ovarian stimulation with TP4/2. 

After 24 h, in the group with TP4/2 treatment, the expression of the *Egr-1* gene was significantly higher than in the group with hCG treatment, which correlated with a lower level of expression of the gene encoding VEGF-A in the FT24 group (Table 6). 

The COX-2 enzyme and prostaglandin E2, the synthesis of which is catalyzed by COX-2, play an important role in maintaining normal oocyte maturation, follicle rupture, and induction of ovulation, and the expression of the *Cox-2* gene is under the control of gonadotropins with LH activity and increases immediately after the ovulatory LH peak [80]. LH and hCG enhance *Cox-2* gene expression by activating the transcription factor CCAAT/enhancer binding protein-α (C/EBPα), which interacts with the *Cox-2* gene promoter. Of decisive importance in this effect of gonadotropins is their stimulating effect on the LH/hCG receptor–G_s_ protein–adenylate cyclase–cAMP–protein kinase A signaling pathway, especially since the effect of LH and hCG is reproduced using non-hydrolyzable cAMP analogs [81]. A significant increase in the expression of the LH/hCG receptor in granulosa cells during the the preovulatory period plays an important role in the control of the expression of the *Cox-2* gene since the weakening of *Lhcgr* gene expression during this period or inactivation of mutations in this gene suppress the gonadotropin-induced peak of COX-2 expression and reduce the level of prostaglandin E2, which interferes with the normal course of ovulation. This is characteristic of endometriosis and the closely associated luteinized unruptured follicle (LUF) syndrome [81]. Suppression of COX-2 expression by knocking out the gene encoding this enzyme, as well as pharmacological antagonists of COX-2, have similar effects, and the administration of exogenous prostaglandin E2 significantly normalizes the ovulation process [82,83,84]. Four hours after treatment with LH/hCG receptor agonists, an early peak in the *Cox-2* gene expression was detected (30–35-fold increase), and the effectiveness of TP4/2 and hCG was comparable. Subsequently, *Cox-2* gene expression began to rapidly decrease, and after 8 h it did not differ from the control (Table 6). 

Matrix metalloproteinase with thrombospondin type 1 motifs-1 (ADAMTS-1) in the ovaries is localized in the cumulus–oocyte complex and carries out the degradation of large-sized aggregating proteoglycans, including versican, which is necessary for the reorganization of the extracellular matrix and the normal course of folliculogenesis and ovulation [85,86]. The expression and functional activity of ADAMTS-1 in cumulus cells are closely related to the ability of the oocyte to fertilize [35], and a decrease in the expression of this metalloproteinase is associated with impaired maturation and quality of oocytes in women with polycystic ovary syndrome [86,87,88,89]. Expression of the gene encoding ADAMTS-1 begins several hours after administration of LH or hCG, as a response to gonadotropin-induced increases in the level of proinflammatory factors, and was elevated until ovulation, after which it decreased during the CL formation [90,91]. 

We have shown that after 4 h, the expression of the *Adamts-1* gene in the ovaries of rats treated with both LH/hCG receptor agonists increases and reaches a maximum after 8 h, when we showed a decrease in the number of preovulatory follicles and an increase in the number of ovulated follicles (Table 2 and Table 6). Along with this, after 8 h, an increase in the amount of ADAMTS protein was shown in the ovaries of rats treated with both LH receptor agonists, which was assessed using an immunochemical assay with antibodies to ADAMTS-1 (Figure 3). The stimulating effects of TP4/2 and hCG on *Adamts-1* expression and the content of ADAMTS-1 in the ovary were comparable, indicating the similarity of their regulatory influence on the production of this metalloproteinase, which is responsible for the dissolution of the connective tissue of the follicle. There is evidence that the level of expression of the *Adamts-1* gene is positively correlated with the production of progesterone, as the main inducer of the expression of this gene [86,92], and this correlates well with our data on a consistent increase in the level of progesterone in the blood and the expression of the *Adamts-1* gene in the ovaries of rats (Table 3 and Table 6). Of importance in this case is the increase in the expression of progesterone receptors in granulosa cells, which is caused not only by the preovulatory LH peak and gonadotropin preparations with LH activity but also by FSH [92,93]. This explains the significant increase in the *Adamts-1* gene expression and content of ADAMTS-1 in the F72 group, without the LH/hCG receptor agonist treatment (Table 6). 

At the stage of formation of the CL, immediately after ovulation, in luteinized granulosa cells there is an increase in the expression of the *Mt-1* gene, encoding metallothionein-1 [33,94]. This protein regulates metalloproteinases, including ADAMTS-1, and also protects ovarian cells from oxidative stress and pro-inflammatory factors, which increase significantly during ovulation [94]. We have shown that the level of expression of the *Mt-1* gene increases 16 h after treatment with both LH/hCG receptor agonists and reaches a maximum after 24 h, and in the case of hCG, the stimulating effect on the expression of this gene is more pronounced than in the case of TP4/2. In the FG24 group, it is higher by 146% in comparison with the FT24 group (Table 6). We believe that this difference is due to a higher level of inflammatory and angiogenic factors in the hCG-treated groups, as we showed when studying the expression of the *Vegf-a* gene (Table 5). 

## 4. Materials and Methods

### 4.1. Experimental Animals and Ethical Standards for Working with Animals

Female Wistar rats *Rattus norvegicus* were obtained from the “Rappolovo” nursery (Leningrad Region, Russia). The animals were housed in plastic cages, six animals in each, with a normal light–dark cycle (12 h/12 h, light on at 9.00 a.m.) and room temperature (22 ± 3 °C), and had free access to standard laboratory chow pellets and drinking water. All experiments were approved by the Bioethics Committee at the Sechenov Institute of Evolutionary Physiology and Biochemistry, St. Petersburg, Russian Academy of Sciences (IEPhB RAS) (protocol #4-1/2023 of 25 April 2023 of the Bioethics Committee of IEPhB RAS, approved by the order of the Director of IEPhB RAS No. 8 of 24 January 2023) and were performed according to the Declaration of Helsinki, “The Guide for the Care and Use of Laboratory Animals” and the European Communities Council Directive of 1986 (86/609/EEC). Every effort was made to minimize the suffering of the animals. 

### 4.2. The Drugs and Biochemical Reagents

The synthesis of 5-amino-N-(*tert*-butyl)-4-(3-(1-methyl-1H-pyrazole-4-carboxamido)phenyl)-2-(methylthio)thieno[2,3-d]pyrimidine-6-carboxamide (TP4/2) was carried out according to the method [95], as described previously by us [24], with minor modifications. For this, 1.1 equivalents of 1-methyl-*1H*-pyrazole-4-carboxylic acid were dissolved in dry *N*,*N*-dimethylformamide and mixed with *N*,*N*-diisopropylethylamine (1.2 equivalents). Then, 1.1 equivalents of 1-[bis(dimethylamino)methylene]-*1H*-1,2,3-triazolo[4,5-*b*]-pyridinium 3-oxide hexafluorophosphate (HATU) was added to the reaction mixture. After 15 min of stirring at room temperature, 5-amino-4-(3-aminophenyl)-*N*-(*tert*-butyl)-2-(methylthio)thieno[2,3-*d*]pyrimidine-6-carboxamide was added, and the reaction mixture was left to stir at the room temperature for the additional 5 h. After the removal of solvents under reduced pressure, the residue was washed with distilled water, and the crude material was filtered off. The target product was purified by column chromatography on silica gel and characterized by high-resolution mass spectrometry (HRMS) and NMR. The high-resolution mass spectra were recorded using a “Bruker micrOTOF” spectrometer (“Bruker”, Hamburg, Germany). The NMR spectra were obtained using a “Bruker Advance III 400” spectrometer (400.13 MHz for 1H and 100.61 MHz for 13C) (“Bruker”, Hamburg, Germany) in DMSO-*d*6 and were referenced to residual solvent proton signal (*δ*H = 2.50) and solvent carbon signal (*δ*C = 39.5). The compound TP4/2 (C_23_H_25_N_7_O_2_S_2_) is a yellow solid with a melting point of 179–181 °C; ^1^H-NMR spectrum (DMSO-*d*6), δ, ppm (*J*, Hz): 10.08 (1H, s, 3′-NH); 8.35 (1H, s, 3″-H); 8.05 (1H, s, 5″-H); 7.94–8.02 (2H, m, 2′-H and 6′-H); 7.56 (1H, t, *J* = 7.8, 5′-H); 7.35 (1H, d, *J* = 7.8, 4′-H); 6.98 (1H, s, NH-*t*-Bu); 6.14 (2H, s, NH_2_); 3.91 (3H, s, N-CH3); 2.62 (3H, s, SCH_3_); 1.37 (9H, s, C(CH3)3); and ^13^C-NMR spectrum (DMSO-*d*6), δ, ppm: 168.8; 167.8; 165.2; 162.7; 161.2; 144.7; 140.0; 139.4; 137.0; 133.3; 129.7; 124.0; 122.1; 120.5; 118.7; 117.8; 97.7; 51.9 (C(CH_3_)_3_); 29.2 (C(CH_3_)_3_); 14.3 (SCH_3_). According to HRMS (ESI-TOF), the found value was 518.1419 (calculated for [M+Na^+^]: 518.1403).

“Follimag” (1000 IU/bottle), a gonadotropin with FSH-like activity isolated from the serum of pregnant mares, was obtained from “Mosagrogen” (Moscow, Russia). Human chorionic gonadotropin (1000 IU/bottle) was purchased from “Moscow Endocrinological Plant” (Moscow, Russia). The rabbit polyclonal Antibody to ADAMTS1 (cat. no. PAB973Ra02) was obtained from “Cloud-Clone Corp.” (Wuhan, China). Normal goat serum (cat. no. S2000) was purchased from the “Biowest” (Paris, France). The peroxidase-conjugated secondary goat anti-rabbit IgG (Cat. No A0545) was obtained from “Sigma” (St. Louis, MO, USA) The other biochemical reagents used in this work were purchased from the company “Sigma-Aldrich” (St. Louis, MO, USA). 

### 4.3. Procedure for Ovulation Induction in Immature Female Wistar Rats

For the experiments, immature female Wistar rats aged 22–24 days were taken. To stimulate folliculogenesis, we used “Follimag”, which was administered subcutaneously (15 IU/rat). At 48 h after treatment with “Follimag”, the animals were treated with TP4/2 (orally, 40 mg/kg) and hCG (s.c., 15 IU/rat) (“Moscow Endocrinological Plant”, Moscow, Russia) (Figure 1). Instead of the study drugs, the control groups received DMSO, a TP4/2 solvent, which was administered orally in the same volume as the TP4/2 solution. Preliminary experiments showed that in the volume used (200 μL), DMSO did not affect the parameters of ovarian steroidogenesis and the induction of ovulation when administered orally. At the end of the experiment (at the time points indicated in Figure 1) immature female rats were anesthetized (chloral hydrate, 400 mg/kg, i.p.) and then decapitated. After decapitation, the left and right ovaries were collected and weighed, the number of tertiary (antral) and preovulatory follicles and CL was assessed using microscopy, and the distribution of ADAMTS-1 was studied via immunohistochemical analysis. The expression of target genes in the ovary was measured using real-time PCR. Along with this, whole blood was collected from the jugular vein of rats to determine the plasma level of steroid hormones (estradiol, progesterone) and LH. 

As a result, 12 groups of animals (*n* = 6 in each) were formed (Figure 1): (1–2) control rats (groups C1 and C2), which instead of “Follimag” were administered its solvent at the same time and were removed from the experiment 48 h (C1) and 72 h (C2) after its start; (3–4) rats that were injected with “Follimag” (15 IU/rat, s.c.) and then withdrawn from the experiment 48 or 72 h after the injection of this drug (groups F48 and F72); (5–8) rats, which were injected with “Follimag”, then treated with hCG (15 IU/rat, s.c.) 48 h later, and decapitated 4, 8, 16, and 24 h after the administration of gonadotropin (groups FG4, FG8, FG16, and FG24, respectively); and (9–12) rats, which were injected with “Follimag”, treated with TP4/2 (40 mg/kg, orally) 48 h later, and decapitated 4, 8, 16, and 24 h after administration of thieno[2,3-d ]-pyrimidine (groups FT4, FT8, FT16, and FT24, respectively). 

### 4.4. Morphological Analysis of the Ovaries

For morphological analysis, the ovaries were fixed for 48 h (+4 °C) in a 4% solution of *para*-formaldehyde (“Sigma”, St. Louis, MO, USA), which was prepared in phosphate buffer (20 mM, pH 7.4) containing 0.9% NaCl (PBS). After washing in PBS, the samples were placed in a 30% sucrose solution in PBS (+4 °C) and, after cryoprotection, frozen on dry ice in Tissue-Tek^®^ medium (“Sacura, Finetek Europe”, Alphen aan den Rijn, The Netherlands). Serial longitudinal sections of the ovary (thickness 10 μm) were obtained using a Leica CM-1520 cryostat (“Leica Microsystems”, Wetzlar, Germany). Every fourth section for histochemical staining was mounted on Super-Frost gelatin-coated glass (“Menzel”, Berlin, Germany), and every tenth section for immunohistochemical analysis was mounted on Super-Frost/plus glass (“Menzel”, Berlin, Germany). Sections from animals of different groups were mounted on the same glass. Gelatin-coated slides, after being washed in PBS and treated with a 50% ethanol solution for 15 min, were stained with Sudan-3 dye (15 min), and then counterstained using Mayer’s hematoxylin according to the standard procedure and placed under a coverslip with glycerol. The resulting images were analyzed in transmitted light using a Carl Zeiss Imager A1 microscope (“Carl Zeiss”, Jena, Germany) with an Axiocam 712 video camera using Zen 3.4 (blue edition) software. Using the ovarian sections, the number of tertiary follicles (6th and 7th follicle types according to the Pedersen and Peters classification), preovulatory follicles (8th follicle type according to the Pedersen and Peters classification), and CL were counted [96,97]. 

### 4.5. Immunohistochemical Analysis of the Distribution of Metalloproteinase ADAMTS-1

The immunohistochemical reaction was carried out according to the protocol, modified in accordance with the results obtained from the evaluation of the negative control (reactions without the primary and secondary antibodies) and recommendations for the implementation of immunohistochemical studies of the ovaries made by other authors [85,98,99]. For immunohistochemical studies, the sections mounted on Super-Frost/plus glasses (“Menzel”, Berlin, Germany) were used. Slides with sections were washed in PBS (20 mM, pH 7.4), after which endogenous peroxidase was blocked for 10 min using a 3% methanol solution of H_2_O_2_. After blocking peroxidase activity, the slides were washed again in PBS and then in PBS containing 0.01% Tween-20 (PBST) (“ChimMed”, Moscow, Russia). Blocking of nonspecific binding was carried out for 1 h in a blocking solution containing 10% normal goat serum (“Biowest”, Paris, France) in PBST. Sections were then incubated overnight at room temperature with rabbit anti-ADAMTS-1 primary antibodies (“Cloud-Clone Corp.”, Wuhan, China) diluted in blocking solution at a ratio of 1:25. After thorough washing in PBST, sections were incubated for 1 h with peroxidase-conjugated secondary goat anti-rabbit IgG (“Sigma”, St. Louis, MO, USA) diluted 1:400 in PBST. Then, the slides with sections were washed in PBS. The reaction was visualized in a solution containing 0.05% diaminobenzidine (“Sigma”, St. Louis, MO, USA) in PBS using 0.015% H_2_O_2_ for 6 min, after which the slides with sections were thoroughly washed in distilled water and placed under a coverslip with glycerol.

### 4.6. Determination of Plasma Levels of Hormones

The levels of estradiol and progesterone in the blood of immature female rats were determined using the Estradiol-ELISA and Progesterone-ELISA kits (“CHEMA”, Moscow, Russia). The level of LH in the blood of animals was determined using the “ELISA for LH, Rat” kit (“Cloud-Clone Corp.”, Houston, TX, USA).

### 4.7. Assessment of Ovarian Gene Expression

Total RNA from the ovaries was isolated using the ExtractRNA reagent (“Evrogen”, Moscow, Russia), and then reverse transcription was performed to obtain cDNA using the MMLV RT Kit (“Evrogen”, Moscow, Russia). Real-time PCR was carried out on an “Applied Biosystems^®^ 7500 Real-Time PCR System” amplifier (“Life Technologies, Thermo Fisher Scientific Inc.”, Waltham, MA, USA) in a mixture containing 0.4 μM forward and reverse primers using the qPCRmix-HS SYBR+Low ROX (“Evrogen”, Moscow, Russia). Table 7 shows the sequences of the primers used to assess the expression of the target genes. We studied the expression of genes encoding the following proteins: (1) cholesterol-transporting protein StAR (*Star*), (2) cytochrome P450_scc_ (side chain cleavage enzyme, CYP11A1), which converts cholesterol into pregnenolone (*Cyp11a1*), (3) cytochrome P450c17 (CYP17A1), catalyzing the conversion of pregnenolone to 17-OH-pregnenolone and then to dehydroepiandrosterone (DHEA) or the conversion of progesterone to 17-OH-progesterone and then to androstenedione (*Cyp17a1*), (4) cytochrome P450c19 (aromatase), catalyzing the conversion of androgens to estrogens (*Cyp19a1*), (5) LH/hCG receptor (*Lhcgr*), (6, 7) the isoforms A and B of vascular endothelial growth factor (*Vegf-a, Vegf-b*), (8) cyclooxygenase-2 (*Cox-2*), (9) early growth response protein-1 (*Egr-1*), (10) matrix metalloproteinase with thrombospondin motifs 1 (ADAMTS-1, *Adamts-1*), and (11) metallothionein-1 (MT-1, *Mt-1*). The obtained data on gene expression were calculated using the delta-delta Ct method and expressed as fold expression relative to expression in the corresponding control group [100]. The expression for the gene *Actb* encoding actin B and the gene *18S rRNA* were used as the endogenous controls. For calculations, we used 7500 Software v2.0.6 and Expression Suite Software v1.0.3. 

### 4.8. Statistical Analysis

Statistical analysis was performed using IBM SPSS Statistics 26 (“IBM”, Armonk, NY, USA). The normality of distribution was tested via the Shapiro–Wilk test, while Livigne’s test was used for the equality of variances. Since most of the data were non-normally distributed (effects of TP4/2 and hCG on ovarian gene expression and plasma hormone levels, and the data on the number of tertiary and preovulatory follicles in morphological investigations), the comparisons between groups were made using the non-parametric median test. Post hoc analysis was performed using Bonferroni’s multiple comparison test. To compare the effects of TP4/2 and hCG at the same time point, the Kruskal–Wallis H test was used, followed by pairwise comparisons using the Mann–Whitney U-test. The data are presented as median and interquartile range (25%; 75%). Under normal distribution conditions, the effects of TP4/2 and hCG treatment on the body weight, ovarian weight, and the ratio of ovarian weight/body weight were performed using one-way ANOVA. Post hoc analysis was performed using Tukey’s test. The data are presented as mean ± standard error of the mean (M ± SEM). All differences are considered as significant at *p <* 0.05. 

## 5. Conclusions

We have shown that compound TP4/2, a thieno[2,3-d]-pyrimidine derivative with the activity of an allosteric agonist of the LH/hCG receptor, induces ovulation when administered orally at a dose of 40 mg/kg to immature female rats pre-treated with FSH. This is due to its steroidogenic effect and the ability to influence the expression of LH-dependent genes involved in the control of late folliculogenesis, ovulation, and CL formation. It was found that TP4/2 increases the number of preovulatory follicles and further stimulates the formation of CL (16 h after treatment), increases the plasma progesterone levels (after 8 h), and time-dependently increases the expression of steroidogenic genes encoding the cholesterol-transporting protein StAR and the cytochromes CYP11A1 and CYP17A1, as well as the expression of genes encoding metalloproteinase ADAMTS-1, cyclooxygenase-2, early growth response protein-1, and metallothionein-1, regulating different stages of late folliculogenesis and ovulation. It is important to note that these effects, both qualitatively and in terms of dynamics, were similar to those of hCG, although in some cases they differed quantitatively, being less pronounced in comparison with hCG-treated rats. At the same time, there were some significant differences between TP4/2 and hCG, and these differences mainly concerned their effects on the expression of genes encoding VEGF-A and LH/hCG receptor in the ovaries and the plasma level of LH. When rats were treated with TP4/2, the plasma LH level did not differ from that in the control, and the expression of the LH/hCG receptor gene in the ovaries, increased in the preovulatory period, was maintained at a high level. In the case of hCG, a decrease in both LH level and *Lhcgr* gene expression was observed. Thus, during treatment with TP4/2, the sensitivity of the ovaries to LH was preserved, and, as can be assumed, the functional activity of the hypothalamic and pituitary parts of the gonadal axis changed to a small extent, which was previously shown by us in the study of thieno[2.3-d]-pyrimidine derivatives on the hypothalamic-pituitary-testicular axis [23,24,25]. Along with this, TP4/2 only transiently (after 4 h) increased the expression of the gene encoding VEGF-A, a risk factor for OHSS development. This is consistent with the less pronounced increase in ovarian size in TP4/2-treated rats compared with hCG-treated groups, and the main reason for this is the more moderate stimulating effect of TP4/2 on ovarian steroidogenesis. It is very important that TP4/2 was active when administered orally, due to its stability and good absorption in the gastrointestinal tract, and this opens up good prospects for creating oral ovulation inducers based on low-molecular-weight allosteric agonists of the LH/hCG receptor, convenient for use in ART. 

## Figures and Tables

**Table 1:** The body weight, total weight of the right and left ovaries, and the ratio of ovary weight/body weight in immature female rats and the effect of treatment with “Follimag” (15 IU/rat, s.c.) and then with LH/hCG receptor agonists, hCG (15 IU/rat, s.c.), and TP4/2 (40 mg/kg, orally).

Rat Group	Body Weight, g	Weight of the Right and Left Ovaries, mg	Ratio of Ovary Weight/Body Weight, %
C1	53.9 ± 1.6	11.5 ± 1.0	0.021 ± 0.002
C2	62.3 ± 1.9	15.0 ± 1.1 ^a^	0.024 ± 0.001
F48	55.4 ± 1.7	26.4 ± 0.9 ^a^	0.048 ± 0.002 ^a^
F72	63.7 ± 2.0 ^a^	27.0 ± 1.4 ^a^	0.043 ± 0.002 ^a^
FG4	57.1 ± 2.1	36.5 ± 2.2 ^a,b,c^	0.064 ± 0.003 ^a,b,c^
FG8	56.1 ± 1.8	39.5 ± 2.3 ^a,b,c^	0.071 ± 0.004 ^a,b,c^
FG16	61.7 ± 1.5	47.3 ± 2.5 ^a,b,c,d^	0.077 ± 0.004 ^a,b,c,d^
FG24	62.1 ± 2.8	43.4 ± 1.5 ^a,b,c^	0.070 ± 0.004 ^a,b,c^
FT4	56.6 ± 1.8	28.8 ± 1.8 ^a^	0.051 ± 0.003 ^a^
FT8	58.4 ± 1.8	31.9 ± 2.0 ^a^	0.055 ± 0.003 ^a,c^
FT16	59.0 ± 2.0	37.4 ± 1.7 ^a,b,c,d^	0.064 ± 0.003 ^a,b,c,d^
FT24	58.6 ± 2.2	39.9 ± 1.2 ^a,b,c,d,e^	0.069 ± 0.003 ^a,b,c,d,e^

Notes. The mass of the ovaries, including when calculating the ratio of ovary weight/body weight, is the sum of the masses of the left and right ovaries. For body weight, ovary weight, and the ovary weight/body weight ratio, the differences with the groups C1 (^a^), F48 (^b^), or F72 (^c^) are significant at *p* < 0.05; ^d^—the differences between the groups FG8, FG16, or FG24 and the group FG4 or between the groups FT8, FT16, or FT24 and the group FT4 are significant at *p* < 0.05; ^e^—the differences between the groups FG24 and FG8 or between the groups FT16 or FT24 and the group FT8 are significant at *p* < 0.05. For details on assessing the significance of differences, see Section 4.8.

**Table 2:** The number of tertiary (antral) and preovulatory follicles, and the number of corpora lutea, in the ovaries of immature female rats, and the effect of treatment with “Follimag” (15 IU/rat, s.c.) and then with LH/hCG receptor agonists, hCG (15 IU/rat, s.c.) and TP4/2 (40 mg/kg, orally).

Rat Group	Tertiary (Antral) Follicles, Units	Preovulatory Follicles, Units	CL, Units
C1	54.5 (48.0; 61.8)	2.0 (0.8; 4.3)	ND
C2	51.5 (41.3; 55.0)	2.0 (2.0; 3.5)	ND
F48	52.5 (47.8; 58.0)	4.5 (4.0; 5.5)	ND
F72	52.5 (45.5; 57.8)	6.5 (5.8; 8.5)	ND
FG4	64.0 (53.0; 73.0)	12.5 (9.0; 18.8) ^a,b^	ND
FG8	50.0 (45.3; 51.3)	4.0 (2.8; 5.3) ^d^	0.3 ± 0.2 (0, 0, 1, 0, 0, 1)
FG16	41.0 (39.3; 45.0)	1.5 (1.0: 4.0) ^c,d^	7.8 ± 1.0 (9, 4, 11, 6, 9, 8)
FG24	32.0 (28.5; 37.5) ^a,b,c,d,e,f^	1.0 (0.0; 2.0) ^b,c,d^	3.5 ± 0.3 (3, 3, 4, 5, 3, 3)
FT4	59.5 (52.5; 69.0)	14.0 (9.8; 19.5) ^a,b^	ND
FT8	46.5 (43.5; 54.0)	4.5 (3.5; 6.3) ^d^	0.2 ± 0.2 (0, 0, 0, 1, 0, 0)
FT16	47.0 (44.5; 52.0)	2.0 (1.0; 3.3) ^c,d^	2.5 ± 0.4 (2, 4, 1, 3, 3, 2) ^#^
FT24	41.5 (35.8; 46.3)	0.0 (0.0; 1.0) ^b,c,d,e^	7.2 ± 0.8 (5, 6, 9, 6, 7, 10) ^#^

Notes. In all cases, the number of tertiary and preovulatory follicles or the number of CL is calculated per ovary. The differences with the groups C1 (^a^), F48 (^b^), or F72 (^c^) are significant at *p* < 0.05; ^d^—the differences between the groups FG8, FG16, or FG24 and the group FG4 or between the groups FT8, FT16, or FT24 and the group FT4 are significant at *p* < 0.05; ^e^—the differences between the groups FG16 or FG24 and the group FG8 or between the groups FT16 or FT24 and the group FT8 are significant at *p* < 0.05; ^f^—the differences between the groups FG16 and FG24 are significant at *p* < 0.05; ^#^—the differences between pairs of groups, such as FG16/FT16 and FG24/FT24, are significant at *p* < 0.05. The data on the tertiary (antral) and preovulatory follicles are presented as the median and interquartile range (25%; 75%). The number of the CL for individual animals (each group contains 6 female rats) is presented in parentheses, and the results are presented as mean ± SEM. For details on assessing the significance of differences, see Section 4.8.

**Table 3:** The concentrations of estradiol, progesterone, and LH in the blood of immature female rats and the effect of treatment with “Follimag” (15 IU/rat, s.c.) and then with LH/hCG receptor agonists, hCG (15 IU/rat, s.c.) and TP4/2 (40 mg/kg, orally).

Rat Group	Estradiol, nmol/L	Progesterone, nmol/L	LH, ng/mL
C1	0.73 (0.66; 0.76)	20.84 (14.69; 23.32)	0.293 (0.249; 0.305)
C2	0.64 (0.43; 0.73)	21.91 (15.71; 27.41)	0.267 (0.248; 0.347)
F48	1.41 (1.10; 1.77) ^a^	19.69 (18.86; 27.50)	0.080 (0.055; 0.087) ^a^
F72	0. 58 (0.47; 0.71)	23.87 (19.15; 29.80)	0.199 (0.114; 0.256) ^b^
FG4	5.01 (2.80; 6.06) ^a,b,c^	18.71 (15.52; 22.39)	0.206 (0.137; 0.362) ^b^
FG8	1.12 (1.00; 1.24) ^a,d^	35.95 (28.97; 38.01) ^a^	0.064 (0.036; 0.102) ^a,d^
FG16	0.75 (0.73; 0.77) ^b,d,e^	62.02 (49.17; 78.90) ^a,b,c,d,e^	0.164 (0.117; 0.205) ^a,b^
FG24	1.01 (0.94; 1,10) ^a,d,f^	12.75 (9.70; 14.29) ^c,f,e^	0.166 (0.134; 0.256) ^b,e^
FT4	4.40 (2.83; 5.30) ^a,b,c^	19.01 (14.82; 20.78)	0.258 (0.228; 0.318) ^b^
FT8	1.65 (1.54; 1.69) ^a,c,d,#^	25.21 (19.69; 27.86) ^#^	0.167 (0.156; 0.277) ^b,#^
FT16	1.15 (1.01; 1.27) ^a,d,#^	40.69 (35.14; 46.76) ^a,b,c,d,e,#^	0.240 (0.183; 0.275) ^b^
FT24	1.22 (0.96; 1.35) ^a,d^	14.09 (13.12; 19.07) ^f^	0.223 (0.190; 0.282) ^b^

Notes. The differences with the groups C1 (^a^), F48 (^b^), or F72 (^c^) are significant at *p* < 0.05; ^d^—the differences between the groups FG8, FG16, or FG24 and the group FG4 or between the groups FT8, FT16, or FT24 and the group FT4 are significant at *p* < 0.05; ^e^—the differences between the groups FG16 or FG24 and the group FG8 or between the groups FT16 and FT8 are significant at *p* < 0.05; ^f^—the differences between the groups FG16 and FG24 or between the groups FT16 and FT24 are significant at *p* < 0.05; ^#^—the differences between pairs of groups, such as FG8/FT8 and FG16/FT16, are significant at *p* < 0.05. For details on assessing the significance of differences, see Section 4.8.

**Table 4:** The expression of genes encoding LH/hCG receptors and steroidogenic proteins in the ovaries of immature female rats and the effect of treatment with “Follimag” (15 IU/rat, s.c.) and then with LH/hCG receptor agonists, hCG (15 IU/rat, s.c.) and TP4/2 (40 mg/kg, orally).

Rat Group	*Lhcgr*	*Star*	*Cyp11a1*	*Cyp17a1*	*Cyp19a1*
C1	0.98 (0.82; 1.19)	1.17 (1.15; 1.21)	0.94 (0.87; 1.30)	0.96 (0.83; 1.32)	0.99 (0.73; 1.29)
C2	1.06 (1.00; 1.08)	1.15 (0.77; 1.40)	0.89 (0.85; ±0.96)	1.07 (1.02; 1.67)	1.04 (0.93; 1.31)
F48	6.97 (6.18; 8.95) ^a^	1.16 (1.05; 1.65)	4.47 (4.11; 5.14) ^a^	1.07 (0.72; 1.16)	1.31 (0.93; 1.62)
F72	5.99 (4.02; 7.77) ^a^	4.41 (2.41; 5.04) ^a,b^	4.82 (2.50; 6.51) ^a^	1.63 (1.17; 1.94)	0.75 (0.60; 0.88)
FG4	8.64 (6.57; 10.45) ^a^	14.53 (12.62; 21.11) ^a,b,c^	14.61 (13.59; 16.60) ^a,b,c^	3.14 (2.11; 3.68) ^a,b^	2.48 (2.27; 3.00) ^a,b,c^
FG8	0.71 (0.45; 1.00) ^b,c,d^	15.92 (14.09; 18.17) ^a,b,c^	7.52 (6.93; 9.31) ^a,b,d^	6.10 (4.81; 7.84) ^a,b,c^	0.13 (0.08; 0.16) ^a,b,c,d^
FG16	0.52 (0.36; 0.76) ^b,c,d^	18.90 (13.20; 20.98) ^a,b,c^	14.46 (11.78; 16.91) ^a,b,c,e^	5.09 (4.29; 6.22) ^a,b,c^	0.13 (0.08; 0.17) ^a,b,c,d^
FG24	0.65 (0.56; 0.73) ^b,c,d^	12.74 (10.14; 12.89) ^a,b,c^	14.02 (12.00; 14.77) ^a,b,c,e^	2.50 (2.15; 2.89) ^a,b,e,f^	0.14 (0.11; 0.23) ^a,b,c,d^
FT4	8.88 (7.79; 9.90) ^a^	15.69 (13.64; 19.81) ^a,b,c^	13.91 (11.67; 15.41) ^a,b,c^	2.43 (1.99; 3.35) ^a,b^	1.73 (1.58; 3.14)
FT8	8.79 (7.94; 11.35) ^a,#^	6.73 (5.02; 9.22) ^a,b,d,#^	9.06 (7.17; 10.23) ^a,b,c^	4.93 (4.37; 7.10) ^a,b,c,d^	0.73 (0.67; 0.85) ^d,#^
FT16	7.84 (6.90; 11.20) ^a,#^	7.42 (6.17; 8.57) ^a,b,d,#^	9.28 (9.06; 9.44) ^a,b,c,d,#^	4.63 (4.30; 5.25) ^a,b,c^	0.93 (0.83; 1.02) ^#^
FT24	5.97 (4.88; 7.12) ^a,#^	3.73 (3.12; 4.34) ^a,b,d,#^	7.69 (7.43; 8.76) ^a,b,c,d,#^	2.84 (1.90; 3.08) ^a,b,e,f^	0.91 (0.66; 1.01) ^d,#^

Notes. The differences with the groups C1 (^a^), F48 (^b^), or F72 (^c^) are significant at *p* < 0.05; ^d^—the differences between the groups FG8, FG16, or FG24 and the group FG4 or between the groups FT8, FT16, or FT24 and the group FT4 are significant at *p* < 0.05; ^e^—the differences between the groups FG16 or FG24 and the group FG8 or between the groups FT24 and FT8 are significant at *p* < 0.05; ^f^—the differences between the groups FG16 and FG24 or between the groups FT16 and FT24 are significant at *p* < 0.05; ^#^—the differences between pairs of groups, such as FG8/FT8, FG16/FT16, and FG24/FT24, are significant at *p* < 0.05. For details on assessing the significance of differences, see Section 4.8.

**Table 5:** The gene expression of vascular endothelial growth factor isoforms in the ovaries of immature female rats and the effect of treatment with “Follimag” (15 IU/rat, s.c.) and then with LH/hCG receptor agonists, hCG (15 IU/rat, s.c.) and TP4/2 (40 mg/kg, orally).

Rat Group	Vegf-a	Vegf-b
C1	1.04 (0.86; 1.31)	0.93 (0.59; 1.34)
C2	1.21 (0.96; 1.34)	0.99 (0.85; 1.20)
F48	0.72 (0.69; 0.86)	0.08 (0.05; 0.17) ^a^
F72	1.38 (0.97; 1.93)	0.07 (0.04; 0,08) ^a^
FG4	3.34 (2.84; 3.60) ^a,b^	0.12 (0.06; 0.22) ^a^
FG8	2.24 (2.09; 2.51) ^a,b^	0.15 (0.08; 0.17) ^a^
FG16	2.70 (2.05; 3.36) ^a,b^	0.10 (0.09, 0.12) ^a^
FG24	1.87 (1.62; 2,12) ^b,d^	0.17 (0.11; 0.25) ^a^
FT4	2.98 (1.91; 3.45) ^b^	0.07 (0.05; 0.08) ^a^
FT8	1.05 (0.95; 1.09) ^d,#^	0.07 (0.06; 0.09) ^a^
FT16	1.16 (0.87; 1.25) ^#^	0.05 (0.04; 0.06) ^a,#^
FT24	1.09 (0.73; 1.18) ^d,#^	0.04 (0.03; 0.05) ^a,e,#^

Notes. The differences with the groups C1 (^a^) and F48 (^b^) are significant at *p* < 0.05; ^d^—the differences between the groups FG24 and FG4 or between the groups FT8 or FT24 and the group FT4 are significant at *p* < 0.05; ^e^—the differences between the groups FT24 and FT8 are significant at *p* < 0.05; ^#^—the differences between pairs of groups, such as FG8/FT8, FG16/FT16, and FG24/FT24, are significant at *p* < 0.05. For details on assessing the significance of differences, see Section 4.8.

**Table 6:** The expression of the genes involved in the regulation of the ovulatory cycle in the ovaries of immature female rats and the effect of treatment with “Follimag” (15 IU/rat, s.c.) and then with LH/hCG receptor agonists, hCG (15 IU/rat, s.c.) and TP4/2 (40 mg/kg, orally).

Rat Group	*Cox-2*	*Mt-1*	*Adamts-1*	*Egr-1*
C1	0.98 (0.52; 1.51)	1.10 (0.90; 1.19)	1.07 (0.73; 1.26)	1.07 (0.86; 1.39)
C2	0.96 (0.61; 1.48)	0.96 (0.83; 1.34)	1.00 (0.73; 1.21)	1.15 (0.84; 1.42)
F48	0.28 (0.22; 0.48)	1.06 (0.96; 1.21)	1.29 (1.06; 1.54)	1.68 (1.61; 2.18)
F72	1.32 (0.66; 1.89)	1.48 (1.21; 1.51)	2.37 (2.22; 3.06) ^a,b^	4.13 (2.30; 5.95) ^a^
FG4	35.90 (26.66; 46.92) ^a,b,c^	1.30 (1.06; 1.63)	2.09 (1.89; 2.29) ^a,b^	44.52 (26.24; 51.72) ^a,b,c^
FG8	1.96 (1.19; 3.88) ^b,d^	2.25 (1.40; 2.51)	5.18 (4.93; 6.43) ^a,b^	2.24 (1.89; 2.42) ^d^
FG16	0.59 (0.54; 0.84) ^d^	5.22 (3.52; 6.32) ^a,b,c,d,e^	2.54 (2.33; 3.19) ^a,b,c,d^	1.79 (1.61; 2.81) ^d^
FG24	0.64 (0.49; 0,95) ^d^	11.16 (10.17; 15.26) ^a,b,c,d,e,f^	1.87 (1.39; 2.71) ^a,b^	2.34 (2.02; 2.42) ^d^
FT4	29.75 (22.45; 35.83) ^a,b,c^	1.99 (1.62; 2.54)	2.73 (2.38; 4.23) ^a,b^	42.49 (29.87; 53.28) ^a,b,c^
FT8	2.51 (1.24; 4.11) ^b,d^	3.09 (2.14; 3.35)	4.92 (3.27; 5.14) ^a,b^	2.58 (1.96; 3.28) ^a,d^
FT16	0.53 (0.47; 0.59) ^d^	3.28 (2.86; 3.35) ^a,b,c^	3.01 (2.70; 3.35) ^a,b^	2.47 (2.22; 2.69) ^d^
FT24	0.35 (0.31; 0.48) ^d,e^	4.54 (3.91; 5.77) ^a,b,c,d,#^	1.81 (1.73; 2.01) ^a,b,e,f^	1.04 (0.94; 1.29) ^c,d,e,#^

Notes. The differences with the groups C1 (^a^), F48 (^b^), or F72 (^c^) are significant at *p* < 0.05; ^d^—the differences between the groups FG8, FG16, or FG24 and the group FG4 or between the groups FT8, FT16, or FT24 and the group FT4 are significant at *p* < 0.05; ^e^—the differences between the groups FG16 or FG24 and the group FG8 or between the groups FT24 and FT8 are significant at *p* < 0.05; ^f^—the differences between the groups FG16 and FG24 or between the groups FT16 and FT24 are significant at *p* < 0.05; ^#^—the differences between the groups FG24 and FT24 are significant at *p* < 0.05. For details on assessing the significance of differences, see Section 4.8.

**Table 7:** The sequences of primers used to assess gene expression in rat ovaries.

Genes	Forward/Reverse Sequence	Product Size (bp)	Annealing Temperature (°C)	Genbank
*StAR*	(For) AAGGCTGGAAGAAGGAAAGC (Rev) CACCTGGCACCACCTTACTT	66	55	NM_031558.3
*Cyp11a1*	(For) TATTCCGCTTTGCCTTTGAG (Rev) CACGATCTCCTCCAACATCC	74	55	NM_017286.3
*Cyp17a1*	(For) CATCCCCCACAAGGCTAAC (Rev) TGTGTCCTTGGGGACAGTAAA	61	55	XM_006231435.3
*Cyp19a1*	(For) GGTATCAGCCTGTCGTGGAC (Rev) AGCCTGTGCATTCTTCCGAT	118	56	NM_017085.2
*Lhcgr*	(For) CTGCGCTGTCCTGGCC (Rev) CGACCTCATTAAGTCCCCTGAA	103	55	NM_012978.1
*Vegf-a*	(For) CACTGGACCCTGGCTTTACT (Rev) GACGTCCATGAACTTCACCA	62	55	NM_001287114.1
*Vegf-b*	(For) GCACAAATCAGATGGTGAGAGA (Rev) CAGGAGATGGTTGATGGCTTAG	101	55	XM_006230911.4
*Cox-2*	(For) ATCAAAGCCTTCGCCACTCA (Rev) ACGGGGCCTTCAAAATGTCT	79	55	NM_017232.4
*Egr-1*	(For) CGTAATCCAAGGGGGTCCAG (Rev) GTGTAAGCTCATCCGAGCGA	196	55	NM_012551.3
*Adamts-1*	(For) CTGCTGCCCTCAGGTGTAAA (Rev) TGAGTGGACTAAAGCTGCGG	187	55	NM_024400.2
*Mt-1*	(For) CTGGCACCACACCTTCTACA (Rev) ATGCTCGGTAGAAAACGGGG	94	55	NM_138826.4
*Actb*	(For) CTGGCACCACACCTTCTACA (Rev) AGGTCTCAAACATGATCTGGGT	125	55	NM_031144.3
*18S rRNA*	(For) GGACACGGACAGGATTGACA (Rev) ACCCACGGAATCGAGAAAGA	50	56	XM_039106097.1

## Data Availability

Data is contained within the article.

## Abbreviations

ADAMTS-1 (gene *Adamts-1*)	matrix metalloproteinase with thrombospondin motifs 1 (A disintegrin and metalloproteinase with thrombospondin motif)
ART	assisted reproductive technology
CL	corpus luteum
COX-2 (gene *Cox-2*)	cyclooxygenase-2
CYP11A1 (gene *Cyp11a1*)	cytochrome P450_scc_
CYP17A1 (gene *Cyp17a1*)	cytochrome P450 17A1/steroid 17α-monooxygenase
CYP19A1 (gene *Cyp19a1*)	cytochrome P450A1 (aromatase)
EGR-1 (gene *Egr-1*)	early growth response protein-1
FSH	follicle-stimulating hormone
GnRH	gonadotropin-releasing hormone
hCG	human chorionic gonadotropin
LH	luteinizing hormone
LH/hCG receptor	luteinizing hormone/human chorionic gonadotropin receptor
MT-1 (gene *Mt-1*)	metallothionein-1
OHSS	ovarian hyperstimulation syndrome
StAR (gene *Star*)	cholesterol-transporting protein (steroidogenic acute regulatory)
TP4/2	5-amino-N-(*tert*-butyl)-4-(3-(1-methyl-1H-pyrazole-4-carboxamido)phenyl)-2-(methylthio)thieno[2,3-d]pyrimidine-6-carboxamide
VEGF	vascular endothelial growth factor, including the isoforms A (VEGF-A) and B (VEGF-B)
